# Exogenous Neural Precursor Cell Transplantation Results in Structural and Functional Recovery in a Hypoxic-Ischemic Hemiplegic Mouse Model

**DOI:** 10.1523/ENEURO.0369-18.2018

**Published:** 2018-12-04

**Authors:** Prakasham Rumajogee, Svetlana Altamentova, Lijun Li, Junyi Li, Jian Wang, Alan Kuurstra, Mohamad Khazaei, Stephanie Beldick, Ravi S. Menon, Derek van der Kooy, Michael G. Fehlings

**Affiliations:** 1 Division of Genetics and Development, Krembil Research Institute, University Health Network, Toronto, ON M5T 2S8, Canada; 2 Centre for Functional and Metabolic Mapping, Robarts Research Institute, the University of Western Ontario, London, ON, N6A 5B7, Canada; 3 Institute of Medical Science, University of Toronto, Toronto, ON M5S 1A8, Canada; 4 Department of Surgery, University of Toronto, Toronto, ON, M5S 1P5, Canada

**Keywords:** Cerebral palsy, hypoxic-ischemia, myelination, neural precursor cells, oligodendrocytes, white matter injury

## Abstract

Cerebral palsy (CP) is a common pediatric neurodevelopmental disorder, frequently resulting in motor and developmental deficits and often accompanied by cognitive impairments. A regular pathobiological hallmark of CP is oligodendrocyte maturation impairment resulting in white matter (WM) injury and reduced axonal myelination. Regeneration therapies based on cell replacement are currently limited, but neural precursor cells (NPCs), as cellular support for myelination, represent a promising regeneration strategy to treat CP, although the transplantation parameters (e.g., timing, dosage, mechanism) remain to be determined. We optimized a hemiplegic mouse model of neonatal hypoxia-ischemia that mirrors the pathobiological hallmarks of CP and transplanted NPCs into the corpus callosum (CC), a major white matter structure impacted in CP patients. The NPCs survived, engrafted, and differentiated morphologically in male and female mice. Histology and MRI showed repair of lesioned structures. Furthermore, electrophysiology revealed functional myelination of the CC (e.g., restoration of conduction velocity), while cylinder and CatWalk tests demonstrated motor recovery of the affected forelimb. Endogenous oligodendrocytes, recruited in the CC following transplantation of exogenous NPCs, are the principal actors in this recovery process. The lack of differentiation of the transplanted NPCs is consistent with enhanced recovery due to an indirect mechanism, such as a trophic and/or “bio-bridge” support mediated by endogenous oligodendrocytes. Our work establishes that transplantation of NPCs represents a viable therapeutic strategy for CP treatment, and that the enhanced recovery is mediated by endogenous oligodendrocytes. This will further our understanding and contribute to the improvement of cellular therapeutic strategies.

## Significance Statement

Cerebral palsy (CP) is one of the most common pediatric neurodevelopmental disorders, affecting >17 million people worldwide. Current treatment options for CP are largely restricted to rehabilitative approaches that alleviate and mitigate symptoms. This paper demonstrates, in a mouse model that mimics several pathobiological and clinical features of CP, that the transplantation of exogenous neural precursor cells (NPCs) represents a viable therapeutic option for the treatment of CP. Exogenous NPCs transplanted into the corpus callosum resulted in repair and regeneration of white matter (WM) injury and functional recovery. Restoration of the WM injury was consistent with an indirect effect mediated by the exogenously transplanted NPCs. Therefore, this work supports the use of stem cells for CP treatment.

## Introduction

Cerebral palsy (CP) is an overarching term encompassing a variety of movement disorders that manifest early in childhood. CP patients often show simultaneous impairments in motor control, cognition, memory, learning, and/or other neurologic functions (Graham et al., 2016). There are numerous underlying causes of CP, including events that occur pre-, peri-, and/or postnatally, which may act independently or be linked together (“multiple-hit hypothesis”). Risk factors for CP include preterm birth, maternal/fetal infection or inflammation, hypoxia/ischemia, and genetic predispositions (Rumajogee et al., 2016).

A common pathologic cause of CP is periventricular leukomalacia (PVL), which mainly results from hypoxia-ischemia and infection/inflammation (Khwaja and Volpe, 2008). The periventricular region is highly vascularized and prone to injury following cerebral blood flow alteration, occasionally leading to hemorrhage (De Reuck, 1971; Volpe, 2009a,b). The unilateral injury to one brain hemisphere by PVL results in hemiplegic CP, characterized by the paralysis (hemiplegia) or weakening (hemiparesis) of one side of the body. PVL frequently affects white matter (WM) structures, resulting in failed myelination of axons connecting different brain regions and leading to cognitive and motor deficiencies (Coq et al., 2016; Back, 2017; Sampaio-Baptista and Johansen-Berg, 2017). WM injury is characterized by astrogliosis, microgliosis, and hypo-myelination, due to oligodendrocyte (OL) maturation disruption (Khwaja and Volpe, 2008; Volpe, 2009a,b; Buser et al., 2012; Back and Miller, 2014).

Despite general advancements in health care research, the incidence of CP remains unchanged (Colver et al., 2014). Current treatment options for CP are limited and include therapeutic hypothermia (Thoresen, 2015), surgical interventions (Graham et al., 2016), and rehabilitative strategies (Faulkner et al., 2013), all of which have demonstrated partial functional improvements in CP patients (McAdams and Juul, 2016). However, no therapies have been effective in repairing the injured WM. From a repair and regeneration perspective, stem cell therapy appears to be a promising strategy. However, the optimal parameters of transplantation (e.g., cell type, location, timing, dose) are yet to be determined.

In this study, we optimized a neonatal hypoxic-ischemic (HI) hemiplegic mouse model based on the Rice–Vannucci model (Rice et al., 1981). The brain injury generated was restricted to the hemisphere ipsilateral to the carotid occlusion, and predominantly affected the subcortical and periventricular WM, striatum/thalamus, hippocampus, and cerebral cortex (Vannucci and Vannucci, 2005). Impaired myelination is a central feature of this model (Clowry et al., 2014; Rumajogee et al., 2016).

After HI, we transplanted exogenous neural precursor cells (NPCs) to examine their ability to repair and regenerate the injured WM and restore function. NPCs are a promising cell type due to their fate-restriction and ability to differentiate into neurons, astrocytes, and OLs. Studies from our lab have shown that transplanted NPCs can differentiate into OLs and promote myelination (Karimi-Abdolrezaee et al., 2010; Ruff et al., 2013b). Therefore, the potential for NPCs to differentiate into myelinating OLs and circumvent hypomyelination observed in CP represents an attractive therapeutic option (Faulkner et al., 2013; Ruff et al., 2013a).

We transplanted NPCs into the corpus callosum (CC), the main interhemispheric commissure of the brain composed of densely bundled WM tracts (Richards et al., 2004), as this structure is known to be significantly damaged in children with CP (Davatzikos et al., 2003; Panigrahy et al., 2005; Kulak et al., 2007; Ho et al., 2013; Andronikou et al., 2015), as well as after neonatal HI in animal models (Sheldon et al., 1996; Skoff et al., 2001; Shrivastava et al., 2012). The CC is more involved in premotor and motor coordination than previously suggested, and impaired somatosensory inputs lead to motor deficiencies (Kulak et al., 2007; Paul et al., 2007; Coq et al., 2016), which supports the CC as a target for regeneration.

We used a range of techniques including histology, immunohistochemistry, MRI, electrophysiology, and behavioral testing, to examine the pathology of the HI model and to explore the potential of exogenously transplanted NPCs to restore areas of WM and neurobehavioral function.

## Materials and Methods

### Animal use

Experimental procedures, animal use and care were approved by the Animal Care Committee at the University Health Network in accordance with the policies and procedures outlined by the Canadian Council of Animal Care. C57Bl/6 mice were housed under controlled conditions (12-h light/dark cycles, 24°C temperature, and fed *ad libitum* with automatic watering). The day of birth was defined as postnatal day (PND) 0.

### Study design

At PND7, male and female mice were subject to either a sham surgery (sham) or hypoxia-ischemia surgery (HI). After surgery, all mice were exposed to hypoxic conditions. At PND21, NPCs were transplanted into sham (sham + NPC) and HI (HI + NPC) mice. Mice that did not receive NPC transplant received vehicle (sham + vehicle and HI + vehicle). Mice were randomly selected from each experimental group from several litters, and a number of outcome measures were performed, including immunostaining and histology, electrophysiology, MRI, and behavioral testing (cylinder rearing test and the CatWalk test).

### Hypoxia-ischemia model, based on the Rice–Vannucci model

**Figure 1.**
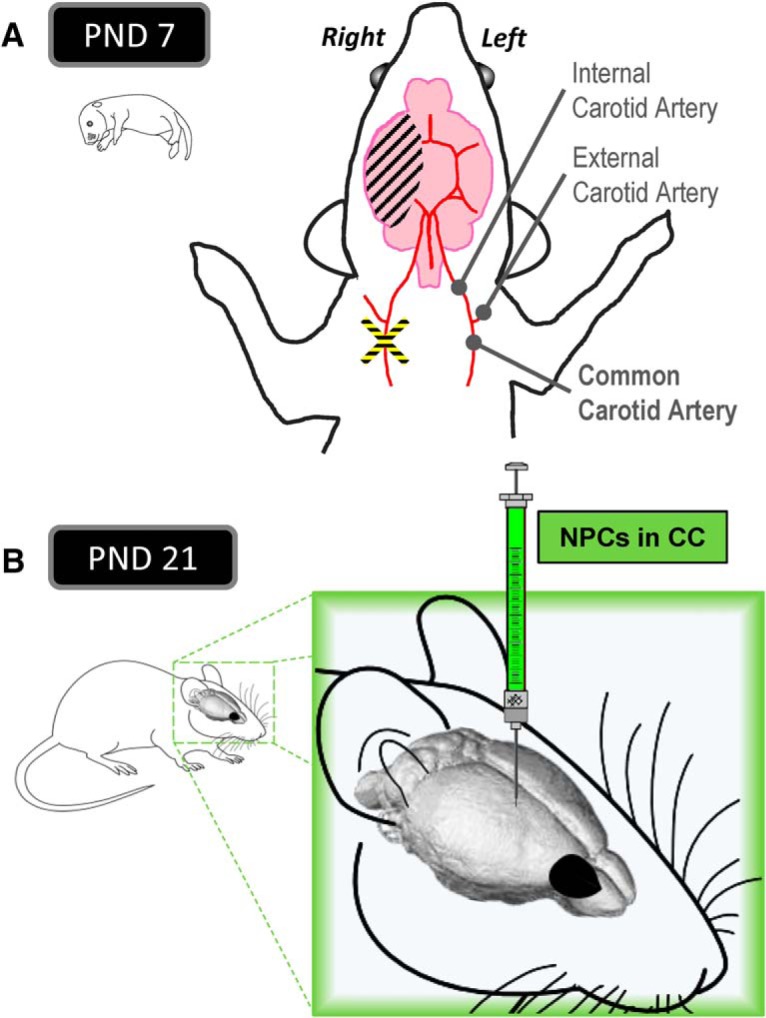
Experimental design. ***A***, The right common carotid artery of postnatal day 7 mice was permanently occluded (ischemia). After 2 h of recovery with the dam, the pup was exposed to 8% O_2_ air (hypoxia). ***B***, At postnatal day 21, NPCs were transplanted in the corpus callosum.

The Rice–Vannucci model is known to generate variable injury, which can be described as mild, moderate, or severe (Rice et al., 1981; Palmer et al., 1990; Balduini et al., 2000; McQuillen et al., 2003; Okusa et al., 2014). In this study, we optimized the experimental conditions by standardizing the surgical procedure and reducing the hypoxia time to obtain a consistent mild HI injury as detailed in Fig. 1. Mild HI injury ensured impairment of the CC without causing complete destruction of the structure and allowed for direct comparisons between mice that did and did not receive NPC transplantation. We used PND7 pups based on histologic similarities with human fetuses at 32–34 weeks of gestation (Johnston et al., 2005; Coq et al., 2016).

#### Ischemia surgery

Each PND7 pup was anaesthetized with a mixture of 5% isoflurane and 95% oxygen for 1.5 min. The pup was then placed in the surgical area where it was maintained under 2.5% isoflurane. An incision was made on the right side of the neck at the shoulder level. For pups in the HI group, the right common carotid artery was isolated from the surrounding tissues, exposed using a fine hook, and permanently cut with a cauterizer (Change-A-Tip, Fine Science Tools). The sham surgery group, which functioned as the control for this experiment, was exposed to the same procedure as the HI surgery group except no carotid occlusion was performed. All pups then received one drop of bupivacaine hydrochloride (Marcaine, 5mg/ml, Hospira/Pfizer) applied to the surgery site as an analgesic. The incision was closed using surgical suture (Sofsilk, Covidien), and the pups recovered under a lamp. The total surgical procedure took 5 min ± 30 s. However, due to the suggestion that isoflurane has a neuroprotective effect (Chen et al., 2011), the pup was kept under 2.5% up to 6.5 min to maintain consistency. The total exposure time was therefore adjusted to 8 min. The pups recovered in a cage under a heating lamp, returned to the dam once all surgeries were completed, and were allowed to recover for 2 h.

#### Hypoxia procedure

After the 2-h recovery period, each pup was placed in a 250-ml ventilated bottle and immersed in a heated water bath (36.5°C). After 15 min of acclimatization, hypoxic air (8% oxygen/92% nitrogen) was delivered and maintained for 45 min (flow rate of 2 l/min). After exposure to the hypoxic environment, the pups were exposed to 15 min of normoxia before they were returned to their dam. The temperature and duration used for this procedure was based on preliminary work demonstrating that longer hypoxia times or higher temperatures led to more severe injury, occasionally producing the total absence of the CC. The death rate was 9%–10% after the hypoxia procedure. Animals (<2%) with a moderate or severe injury (no visible CC; >30% liquefied cavity) were excluded from the study.

### Adult NPC transplantation

Adult NPCs were isolated from transgenic adult mice expressing yellow fluorescent protein (YFP) [strain 129-Tg(ACTB-EYFP)2Nagy/J; The Jackson Laboratory] according to a previously described protocol (Tropepe et al., 1999). Briefly, the subependymal zone of the mouse forebrain was dissected and processed. Cells were dissociated and cultured in uncoated flasks. The nonadherent neurospheres were passaged weekly for 3–6 passages. NPCs were prepared in serum-free medium at a density of 5 × 10^4^ live cells/μl.

NPCs were transplanted in the CC of PND21 mice, which corresponds to a 2-yr-old child from a developmental perspective (Semple et al., 2013). In line with past studies, the injury is already observed at P21. Lesions are already noticeable as soon as 50 h after insult (Rice et al., 1981; Johnston et al., 2005; Vannucci and Vannucci, 2005; Hagberg et al., 2015; Rumajogee et al., 2016). Starting 2 d before transplantation (PND19), Sandimmune was given subcutaneously twice daily (10 mg/kg, SandimmuneI.V., Cyclosporine concentrate, 50 mg/ml, Novartis). For the sham and HI + NPC groups, mice at PND21 were anaesthetized with isoflurane (5% for 90 s), then weighed and shaved. Next, the mice were maintained under 2.5% isoflurane in a stereotaxic frame (David Kopf Instruments) while the transplantation sites were prepared. The skin was disinfected, and an incision was made above the midline. The skull was cleaned with a sterile saline solution, and the Bregma point was located for use as an anatomic reference. Using Mastercarver Micro-Pro, two holes were drilled on the right (ipsilateral injury) side (+1 mm lateral/rostral and +1 mm lateral/caudal), and the meninges were punctured with a fine needle. After the puncture, isoflurane was lowered to 1.5%. Pulled glass needles, mounted on a 5-μl syringe (Microliter Syringe, Hamilton) connected to a micro-pump system (Micro4, World Precision Instruments), were used to inject 2.5 μl cell suspension into the CC (anterior site 1-mm depth; posterior site 1-mm depth/10° angle) at a rate of 250 nl/min. A waiting period of 1 min before injection and 2 min after injection was found to reduce the potential for leaks. After this procedure, the skin was sutured and each mouse was given subcutaneously 0.1 ml of 0.9% saline solution, 0.1 ml buprenorphine (0.048 mg/kg, Comparative Medicine and Animal Resources Centre, McGill University), and Sandimmune (10 mg/kg).

The pups were weaned, and food supplement was added to the cage (Kitten Milk Replacer; PetAg). For the following 2 d, animals were given buprenorphine and Sandimmune twice daily subcutaneously. Afterward, mice were given Cyclosporine in drinking water (0.08 mg/ml; 20 mg/ml solution, Chiron Compounding Pharmacy) and were frequently monitored.

### Structural outcomes

#### Tissue preparation

At specific time points (4 d and 2, 4, 6, 9, 11, 13, and 19 weeks), a subsection of animals was deeply anaesthetized and transcardially perfused with 0.1 M PBS followed by 4% paraformaldehyde/0.1 M PBS, pH 7.4. Brains were then extracted, postfixed for 4 h, cryoprotected, embedded, and frozen as previously described (Ruff et al., 2013b). 20-μm coronal sections from bregma –2.5 to +1.2 mm were collected on Superfrost+ Slides (Fisher Scientific) on the cryostat and stored again at –80°C for later use.

#### Immunostaining and histology

Frozen sections were thawed in double-distilled water, spread using fine brushes, and air-dried before being rehydrated in PBS. For immunostaining, a blocking step was performed for 1 h at room temperature (RT) in a solution of 2% fetal bovine serum and 0.1% Triton X-100 in 0.1 M PBS. The primary antibodies used were mouse anti-MBP (1:1000, Covance, SMI-99P) for myelin; mouse anti-GFAP (1:1000, Millipore, MAB3402) for astrocytes; rabbit anti-Olig2 (1:400; Millipore, Ab9610) for oligodendrocytes; mouse anti-APC (CC1, 1:250, Abcam, Ab16794) for mature oligodendrocytes and astrocytes; Cy3-conjugate mouse anti-Nestin (1:500, Millipore, MAB353C3) as an NPC marker; mouse anti-NeuN (1:500, Millipore, MAB377) for neurons; guinea-pig anti-Doublecortin (DCX, 1:400, Millipore, AB2253) for immature neurons; and mouse anti-chondroitin sulfate (CS-56, 1:1000, Abcam, ab11570) for chondroitin sulfate proteoglycan sulfated in position 6. The brain sections were incubated overnight at 4°C with the primary antibodies diluted in a blocking solution. Double-staining was done using 2 primary antibodies with no cross-reaction. The following day, the sections were washed three times in 0.1 M PBS (washes of 5, 10, and 15 min at RT) and incubated with the suitable secondary antibodies (1:400 in 0.1 M PBS for 1 h at RT): Alexa Fluor 568 IgG (Life Technologies, anti-rabbit A11011; anti-mouse A11031; anti-chicken, A11041) and Alexa Fluor 647 IgG (Life Technologies; anti-mouse, A21235; anti-rabbit, A21244; anti-guinea-pig, A21450). DAPI (1:1000, Life Technologies, D3571) was also used to stain the A-T rich DNA regions. The sections were then washed three times in 0.1 M PBS (washes of 5, 10, and 15 min at RT). Slides were mounted in Mowiol (*Calbiochem*), covered with coverslips, and kept at 4°C. For histologic analysis, Luxol fast blue (LFB) and hematoxylin and eosin (H&E) staining was performed as previously described (Goto, 1987).

#### Quantification

Researchers that participated in the quantification of structures were blinded to the identity of the mice. Eight sham mice, 15 HI + vehicle mice, and 16 HI + NPC mice were included in these analyses.

Stereological assessments of the CC volume, as well as the transplanted NPCs (YFP^+^) and the Olig2^+^ cells counts, were done using the Cavalieri estimator (Stereo Investigator, MBF Bioscience). The transplanted NPCs were counted within the whole injection site in the CC. The Olig2^+^ cells were counted within the CC in a virtual coronal slice of ∼3-mm thickness, which covers both anterior and posterior transplantation sites (∼24 sections). The YFP^+^/Olig2^+^ were also evaluated, as well as the area and volume of the CC.

Mature (Olig2^+^/CC1^+^) and immature (Olig2^+^/CC1^–^) oligodendrocytes were counted in the CC. Three pictures per animal were taken using a confocal microscope (magnification 20×) from 12–15 coronal sections covering both transplantation sites (±1 mm rostrocaudally from bregma). Similarly, the counting of YFP^+^ cells (NPC) which were also positive for GFAP, DCX, NeuN^+^, or Nestin was performed on 6–9 coronal sections per transplanted sham and HI animal.

Additionally, neurons (NeuN^+^) were counted in the cortex. One picture of the primary motor cortex (PMC) and two pictures of the somatosensory cortex (SSC) of each hemisphere were taken from 9–12 coronal sections per animal. Landmarks were used to maintain consistency between coronal sections. The thickness of the PMC and SSC were also assessed. The brain hemispheres and hippocampus areas were evaluated, using Stereo Investigator, on 18 coronal LFB/H&E brain sections per animal, which provided additional data for cortex thickness and CC area.

#### Electrophysiology

Before decapitation and dissection of the brain, mice were deeply anesthetized with sodium pentobarbital (60 mg/kg, i.p.) and underwent transcardiac infusion with cold 95% O_2_/5% CO_2_ saturated sucrose-substituted artificial cerebrospinal fluid (aCSF). aCSF contained (in mM): sucrose 210; NaHCO_3_ 26; KCl 2.5; CaCl_2_ 1; MgCl_2_ 4; NaH_2_PO_4_ 1.25; and d-glucose 10. Eight serial 400-μm-thick coronal slices of the brain containing the CC were obtained on a Leica vibrating microtome VT1200S (Fig. 9*A*; Li et al., 2016). Isolated CCs were severed at the midpoint and separated into left and right halves. These separated CCs were incubated in identifiable positions on a mesh in oxygenated aCSF containing (in mM): NaCl 125; NaHCO_3_ 26; KCl 2.5; CaCl_2_ 2; MgSO_4_ 1.3; NaH_2_PO_4_ 1.25; and d-glucose 10, at RT with 95% O_2_-5% CO_2_ for at least 1 h before electrophysiological recording.

Compound action potentials (CAPs) were acquired using suction electrodes for both stimulating and recording, as previously described (Li et al., 2016). CAPs recorded from the CC provided the information regarding myelinated axons as demonstrated by the fast conduction velocity (CV) first peak (peak1). In contrast, nonmyelinated axons were assessed using the CV of the second peak (peak2). The recordings were performed in a 0.5-ml bath with a 2.8-mm distance between two electrodes continuously perfused at 1 ml/min with aCSF oxygenated by 95% O_2_/5% CO_2_ (Fig. 9*B*). The stimulating pulses of 0.1-ms duration and varying amplitude (0.01∼1.5 mA) were applied via the PSIU6 stimulus isolation unit Grass S88 dual-channel stimulator (Grass Technologies). CAPs were recorded with an Axoprobe 1A amplifier (Molecular Devices/Molecular Devices). The signals were processed using pClamp8 software and DigiData 1320A at a 100-kHz sampling rate (Molecular Devices/Molecular Devices). Data analysis was performed offline using Clampfit10 after filtering the signals with a 10-kHz low-pass filter.

#### Magnetic resonance imaging

MRI was performed on 6 sham mice, 11 HI + vehicle mice and 10 HI + NPC mice. Mice were imaged on a 9.4-T small animal MRI scanner equipped with a 30-mm millipede volume coil (Agilent, Palo Alto). High-resolution *in vivo* anatomic images were obtained using a 3D True-FISP pulse sequence (FOV: 16 × 16 × 16 mm^3^; data matrix: 128 × 128 × 128; TR/TE: 6.0/3.0 ms; flip angle: 30°) yielding an isotropic 125 μm 3D image. Image processing was performed using Advanced Normalization Tools (ANTs) and the Insight Segmentation and Registration Toolkit (ITK). Intensity inhomogeneity was corrected using the N4 algorithm (Tustison et al., 2010), and intensity normalization was performed by histogram matching (Nyul et al., 2000). Images were smoothed by a curvature driven flow algorithm (50 steps of step size 0.0001 were performed on ITK’s finite difference solver), allowing sharp ventricle boundaries to be preserved (Sethian and Sethian, 1999). The ventricle volume was then measured using a connected threshold region growing algorithm (Johnson et al., 2017).

### Functional outcomes

Eight sham mice, 15 HI + vehicle mice, and 16 HI + NPC mice completed the functional tests detailed below. The researchers who performed the functional assessments were blinded to the identity of the mice.

#### The cylinder test

This cylinder test was used to assess preferential forelimb use. Each mouse was placed in a clear plastic cylinder and its rearing activity was recorded for five consecutive minutes with a camera placed beneath the apparatus. The placement of the whole palm on the cylinder wall demonstrates the use of that forelimb for body support. The use of the right (R) unaffected versus left (L) affected forelimb was calculated as a percentage of total contacts: R/(R + L) × 100. Additionally, the forelimb used first in a rearing sequence was calculated.

#### The CatWalk test

The CatWalkXT (v10.6, Noldus Information Technology) was employed to measure walking performance. Each animal walked freely through a corridor on a glass walkway illuminated with beams of light from below. A successful walking trial was defined as having the animal walk at a steady speed (no stopping, rearing, or grooming), and three to five successful trials were collected per animal. The footprints were recorded using a camera positioned below the walkway, and footprint classification was manually corrected to ensure accurate readings (Chen et al., 2017). CatWalkXT software was then used to analyze several gait parameters including swing speed (cm/s), stride length (cm), and paw intensity (arbitrary units, 0–255).

### Statistical analysis

For quantification, the value of the left contralateral side was normalized to 100%. All statistical analyses were performed using GraphPad Prism v6.01. A D’Agostino–Pearson omnibus normality test was performed. For cell survival, an unpaired *t* test was used. For NPC differentiation, a 1-way ANOVA/Kruskal–Wallis test was used. For electrophysiology, a 1*-*way ANOVA was used. For all other outcome measures we used a 2-way ANOVA followed by a Bonferroni *post hoc* test. *F* values, exact *t* values, and when available, exact *p* values are reported. Values reported are mean ± SD. n.s. = not significant; *p < 0.05; **p < 0.01; ***p < 0.001.

## Results

Our model of mild HI injury produced consistent structural changes in key brain regions and deficiencies in functional outcomes. NPC transplantation of exogenous NPCs into HI mice resulted in restoration of brain structures and motor performance.

### Transplanted NPCs survive, migrate, differentiate morphologically, and integrate in the corpus callosum

**Figure 2.**
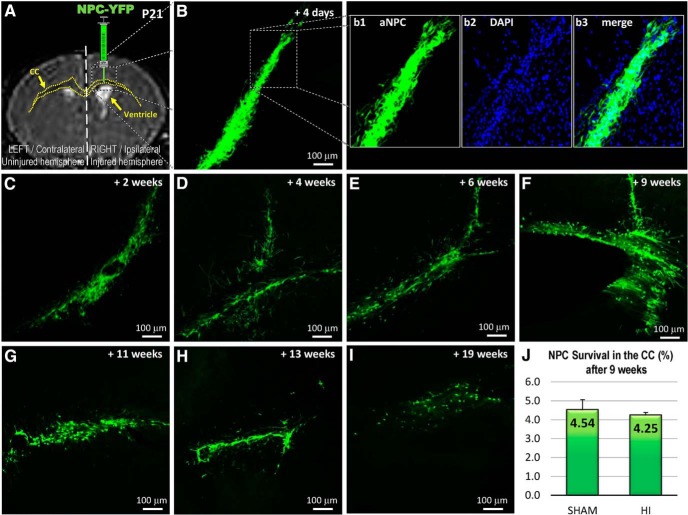
Transplanted NPCs survive, migrate, differentiate morphologically, and integrate within the corpus callosum. At P21, injury is visible by MRI and includes the thinning of the CC (outlined in yellow) and a right ventriculomegaly. ***A***, NPCs were transplanted in the right/ipsilateral/injured CC at PND21. ***B–I***, The NPCs migrated and spread through the CC. Their morphology changed from a round-type (*in vitro*) to a fibroblast-like cell type and, subsequently, to a cell with multiple processes. ***I***, Transplanted NPCs (green) are counterstained by DAPI (blue; b1–b3). The NPCs survived >19 weeks after transplantation. ***J***, The survival rate was similar in the HI and sham animal after 9 weeks.

NPCs transplanted at PND21 into the injured CC (Fig. 2*A*) survived for up to 19 weeks (Fig. 2*B–I*). After 9 weeks, the survival rate between HI (4.25 ± 0.79%) and sham (4.53 ± 0.53%) animals was similar (unpaired *t* test, *p* = 0.6586; Fig. 2*J*). Transplanted NPCs migrated through the CC, colonized the injury site, and engrafted in the brain tissue, with an initial change in shape at day 4 (PND25) from a round-type cell (*in vitro*) to a fibroblast like cell (Fig. 2*B*). Afterward, the NPCs grew numerous processes into the neighboring brain structures and established a network (Fig. 2*C–I*), suggesting some level of communication with neighboring brain structures such as the hippocampus, cortex, and internal capsule. Similar observations were made in the sham (data not shown).

**Figure 3.**
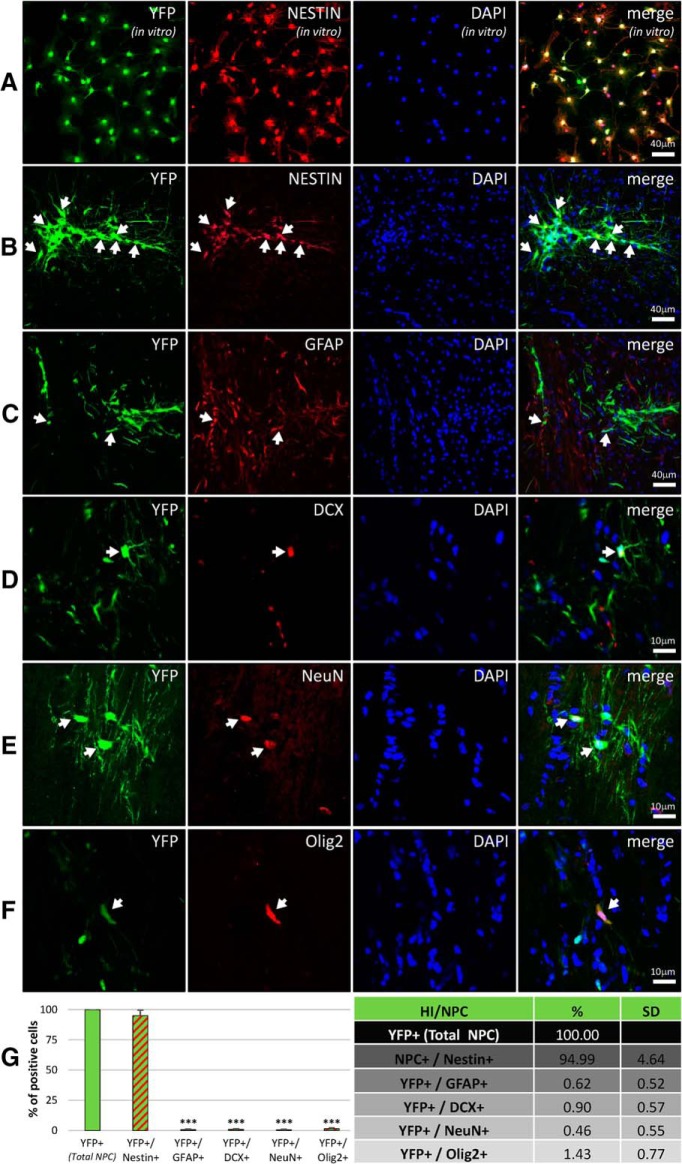
Some transplanted NPCs differentiate in the corpus callosum. ***A***, *In vitro*, all NPCs (YFP^+^) were also Nestin^+^. ***B–F***, Nine weeks after transplantation, the majority of transplanted NPCs (YFP^+^) were also Nestin^+^ (***B***), while a few transplanted NPCs converted into different cell types: astrocytes (GFAP^+^, ***C***), immature neurons (DCX^+^, ***D***), neurons (NeuN^+^, ***E***) or oligodendrocytes (Olig2^+^, ***F***). The vast majority of transplanted NPCs (>94%) were negative for GFAP, DCX, NeuN, or Olig2. ***G***, After transplantation in sham or HI mice, <1% of NPCs differentiated to GFAP^+^, DCX^+^ or NeuN^+^ cells, and <2% differentiated to Olig2^+^ cells, while >94% were Nestin^+^. ***p < 0.001.

Cultured NPCs (YFP^+^) grown *in vitro* stained positive for Nestin before transplantation (Fig. 3*A*). At 9 weeks posttransplantation, the vast majority of NPCs located within the lesioned CC expressed Nestin (94.99 ± 4.64%; Kruskal–Wallis test; *p* = 0.8980; Figs. 3*B* and 2*G*), with very few NPCs differentiating into astrocytes (GFAP^+^, 0.62 ± 0.52%, Kruskal–Wallis test; *p* < 0.0001; Figs. 3*C* and 2*G*), immature neurons (DCX^+^, 0.90 ± 0.57%, Kruskal–Wallis test; *p* < 0.0001; Figs. 3*D* and 2*G*), neurons (NeuN^+^, 0.46 ± 0.55%, Kruskal–Wallis test; *p* < 0.0001; Figs. 3*E* and 2*G*), or oligodendrocytes (Olig2^+^, 1.43 ± 0.77%, Kruskal–Wallis test; *p* = 0.0002; Figs. 3*F* and 2*G*). When transplanted in the sham, the differentiation profile of NPCs was comparable. At 19 weeks posttransplantation, the proportion of cells differentiated into different cell types did not change compared to week 9 (data not shown).

### Transplanted NPCs lead to structural recovery at 9 weeks posttransplantation

**Figure 4.**
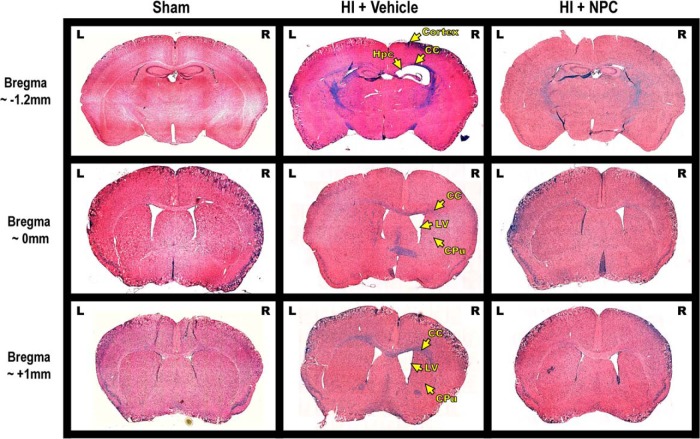
A unilateral injury is generated in different brain structures which, to various extents, are recovered after NPC transplantation. Representative pictures of LFB/H&E brain coronal sections of sham (left column), HI + vehicle (middle column) and HI + NPCs (right column) at 3 different bregma levels that encompass the injury site [–1.2 mm (first line), 0 mm (second line), and +1 mm (third line)]. After neonatal HI, brain lesions were observed in several structures of the right ipsilateral hemisphere, such as the corpus callosum (CC), the cortex, the hippocampus (Hpc), the lateral ventricle (LV), or the caudoputamen (CPu). Various levels of recovery were observed after NPC transplantation in the CC.

Our model of HI injury affects the brain structure, and NPC transplantation produces a substantial level of recovery. Representative pictures of brain coronal sections at three bregma levels show that the neonatal unilateral HI lesion occurs in the right ipsilateral brain hemisphere. Various structures such as the CC, the cortex, the hippocampus, the lateral ventricle, or the caudoputamen are affected (Fig. 4, middle column, arrows) compared to the sham control (Fig. 4, left column). Recovery is observed after NPCs are transplanted in the CC (Fig. 4, right column).

#### HI injury reduces the overall brain size, but NPC transplantation does not restore it

**Figure 5.**
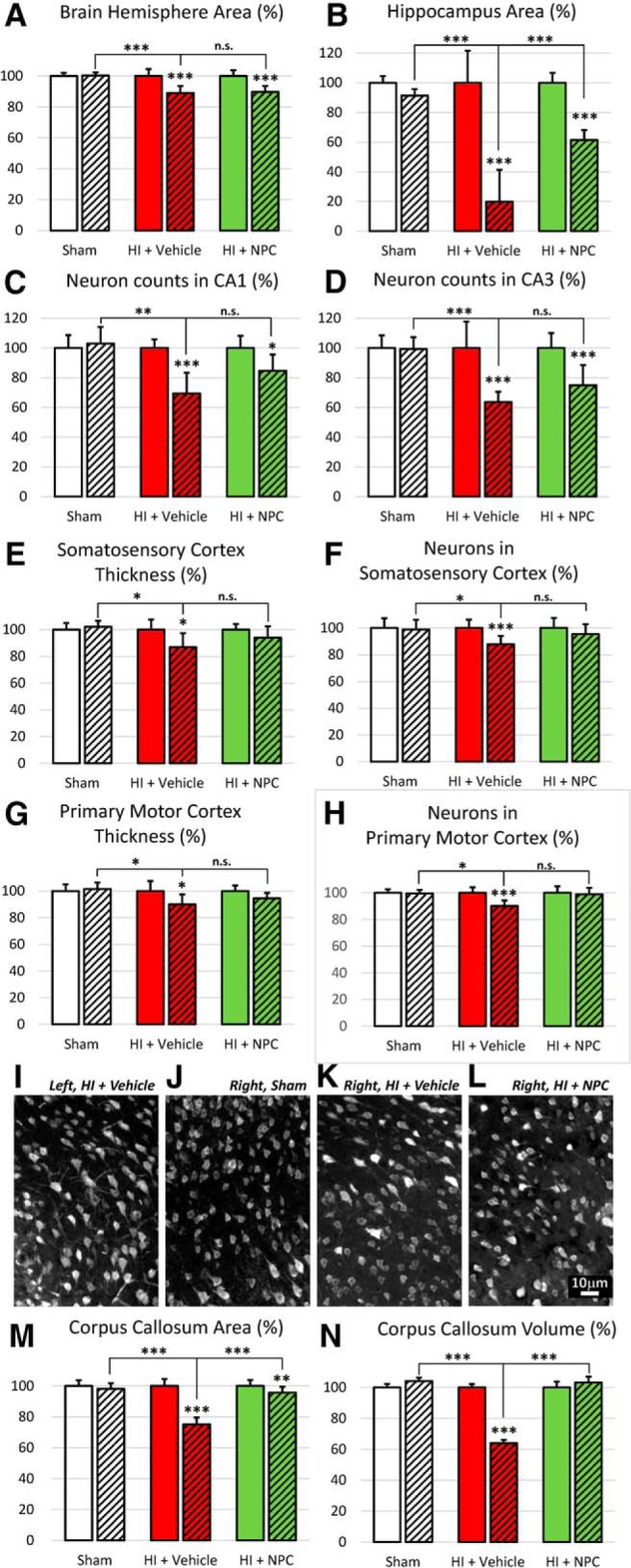
Transplanted NPCs restore the integrity of various structures of the brain, including the corpus callosum. Various brain structures were affected by the HI injury. ***A***, The size of the right brain hemisphere was decreased after HI injury, which was not significantly corrected after NPC transplantation. ***B***, The hippocampus was also affected by the HI injury, and NPC transplantation had only a limited impact on recovery. ***C***, ***D***, After HI injury, the neuron population was also decreased in the CA1 (***C***) and CA3 (***D***) regions, and the NPCs had a limited effect on recovery. ***E***, ***F***, The thickness of the somatosensory cortex (SSC; ***E***) and the primary motor cortex (PMC; ***F***) were decreased after HI injury; the NPCs had a limited effect on recovery. ***G***, ***H***, The NeuN^+^ neuron population was decreased in the SSC (***G***) and in the PMC (***H***) after HI injury, and the NPCs had a limited effect on recovery. ***I–L***, Representative pictures of NeuN^+^ neurons in the SSC of the left/control side of a HI brain (***I***), the right side of the sham brain (***J***), the right/injured side of the HI brain (***K***), and the right/injured side of the HI brain treated with NPCs (***L***). ***M***, The HI injury also led to the decrease of the CC area assessed on coronal sections. ***N***, Stereological analysis confirmed the impact on the CC with a volume decrease. After NPC transplantation, recovery was observed. The left side has been normalized to 100% and is compared with the right side. The dashed versus plain bars represent the right versus left side, respectively. *p < 0.05, **p < 0.01, ***p < 0.001.

After HI injury, the brain size was affected, with the right injured hemisphere being significantly smaller (89.06 ± 4.50%, 2-way ANOVA, followed by Bonferroni’s test, *F*(2, 208) = 52.21) compared to both the uninjured contralateral hemisphere (normalized at 100, *p* < 0.0001, *t* = 11.70) and the ipsilateral hemisphere of the sham animals (99.84 ± 5.13%, *p* < 0.0001, *t* = 13.03; Fig. 5*A*). After NPCs were transplanted in the CC of the HI injured animals, the size of the smaller right brain hemisphere persisted (89.70 ± 4.57%, *p* > 0.9999, *t* = 0.8597; Fig. 5*A*). The transplanted NPCs do not have a clear impact on the overall brain size reduction.

#### HI injury significantly affects the hippocampus, and NPC transplantation leads to limited recovery

In HI injured animals, the right hippocampus was dramatically smaller (19.73 ± 21.63%, 2-way ANOVA, followed by Bonferroni’s test, *F*(2, 46) = 41.08) compared to the uninjured contralateral hemisphere (normalized at 100, *p* < 0.0001, *t* = 12.28) or the ipsilateral hemisphere of the sham mice (91.35 ± 4.51%, *p* < 0.0001, *t* = 12.00; Fig. 5*B*). After NPC transplantation in the CC, the right hippocampus size increased notably (61.49 ± 6.68%), although the size remained smaller compared to the HI contralateral hippocampus (normalized at 100, *p* < 0.0001, *t* = 7.98) and to the sham ipsilateral hippocampus (91.35 ± 4.51%, *p* < 0.0001, *t* = 5.87; Fig. 5*B*).

The neuronal population was also reduced following HI injury, with the number of NeuN^+^ cells being dramatically smaller in the ipsilateral CA1 region of animals having received the vehicle (69.41 ± 13.99%, ANOVA, followed by Bonferroni’s test, *F*(2, 56) = 24.75) compared to the uninjured contralateral CA1 (normalized at 100, *p* < 0.0001, *t* = 8.93) or to the ipsilateral CA1 of the control animals (102.94 ± 11.20%, *p* < 0.0001, *t* = 9.78; Fig. 5*C*). After NPC transplantation, the recovery of the neuron population of the ipsilateral CA1 was limited (84.62 ± 10.97%) compared to the ipsilateral side of the HI + vehicle group (69.41 ± 13.99%, *p* < 0.0005, *t* = 4.54; Fig. 5*C*).

Similarly, the number of NeuN^+^ cells in the ipsilateral CA3 region was lower (63.65 ± 6.90%, 2-way ANOVA, followed by Bonferroni’s test, *F*(2, 56) = 16.92) compared to the uninjured contralateral CA3 (normalized at 100, *p* < 0.0001, *t* = 8.07) or to the ipsilateral CA3 of the control animals (99.33 ± 7.93%, *p* < 0.0001, *t* = 7.92; Fig. 5*D*). The recovery was not significant following NPC transplantation (74.92 ± 13.58%) compared to the HI + vehicle group (63.65 ± 6.90%, *p* = 0.1970, *t* = 2.56; Fig. 5*D*). The transplanted NPCs led to a substantial, although not full, recovery of the hippocampus.

#### HI injury significantly affects the cortex, and NPC transplantation produces significant recovery

The ipsilateral somatosensory cortex (SSC) was thinner (86.83 ± 10.45%, 2-way ANOVA, followed by Bonferroni’s test, *F*(2, 38) = 1.77) compared to the contralateral side (normalized at 100, *p* = 0.02, *t* = 3.44) or to the ipsilateral side of sham animals (102.04 ± 4.48%, *p* = 0.25, *t* = 2.51; Fig. 5*E*). After NPC transplantation, the thickness of the SSC was restored (93.91 ± 8.47%) compared to the contralateral side (normalized at 100, *p* > 0.9999, *t* = 1.22) or the ipsilateral side of sham animals (102.04 ± 4.48%, *p* > 0.9999, *t* = 1.26; Fig. 5*E*).

A significant decrease in the neuronal population was observed after HI injury in ipsilateral SSC (87.75 ± 6.18%, 2-way ANOVA, followed by Bonferroni’s test, *F*(2, 68) = 3.74) compared to the contralateral control side (normalized at 100, *p* = 0.0005, *t* = 4.46) or the ipsilateral side of sham animals (98.88 ± 7.22%, *p* = 0.0111, *t* = 3.54; Fig. 5*F*). After NPC treatment, the neuron population was restored in the SSC (95.34 ± 7.51%) compared with the contralateral side (normalized at 100, *p* = 0.9636, *t* = 1.88) and the ipsilateral side of the sham (98.88 ± 7.22%, *p* > 0.9999, *t* = 1.17; Fig. 5*F*).

Similarly, the ipsilateral primary motor cortex (PMC) was thinner (89.97 ± 7.46%, 2-way ANOVA, followed by Bonferroni’s test, *F*(2, 38) = 2.23) compared to the contralateral side (normalized at 100, *p* = 0.0062, *t* = 3.87) or to the ipsilateral side of sham animals (101.46 ± 4.91%, *p* = 0.12, *t* = 2.81; Fig. 5*G*). After NPC transplantation, the thickness of the PMC was restored (94.49 ± 4.17%) compared to the contralateral side (normalized at 100, *p* > 0.9999, *t* = 1.63) or the ipsilateral side of sham animals (101.46 ± 4.91%, *p* > 0.9999, *t* = 1.59; Fig. 5*G*).

A significant decrease in the neuronal population was observed after HI injury in ipsilateral PMC (90.11 ± 4.12%, 2-way ANOVA, followed by Bonferroni’s test, *F*(2, 68) = 8.85; Fig. 5*H*) compared to the contralateral control side (normalized at 100, *p* < 0.0001, *t* = 5.99) or the ipsilateral side of sham animals (99.57 ± 2.59%, *p* < 0.0001, *t* = 5.00; Fig. 5*H*). After NPC treatment, the neuron population was restored in the PMC (98.81 ± 4.84%) compared with the contralateral side (normalized at 100, *p* > 0.9999, *t* = 0.80) and the ipsilateral side of the sham (99.57 ± 2.59%, *p* > 0.9999, *t* = 0.42; Fig. 5*H*).

The decreased neuron population in the cortex is shown in representative pictures taken in the SSC: NeuN^+^ neurons in the left/control side of an HI brain (Fig. 5*I*), the right side of the sham brain (Fig. 5*J*), the right/injured side of the HI brain (Fig. 5*K*) and the right/injured side of the HI brain treated with NPCs (Fig. 5*L*). The transplanted NPCs generate a significant recovery of the cortex structure and neuronal population.

#### HI injury significantly affects the corpus callosum, and transplanted NPCs lead to recovery

The areas assessed on anterior and posterior coronal sections (Fig. 5*M*) show a significant decrease of the right injured CC area (75.04 ± 4.48%, 2-way ANOVA, followed by Bonferroni’s test, *F*(2, 154) = 117.0) compared to the uninjured contralateral CC (normalized at 100, *p* < 0.0001, *t* = 17.37) or to the ipsilateral CC of the control animals (98.08 ± 3.67%, *p* < 0.0001, *t* = 17.37). After NPC transplantation in the CC, the area of the right CC (95.58 ± 5.88%) was markedly restored compared to the vehicle group (75.04 ± 4.48%, *p* < 0.0001, *t* = 17.73) and became similar to control values (contralateral normalized at 100: *p* < 0.05, *t* = 5.44; and ipsilateral sham: 97.21 ± 4.62%, *p* > 0.9999, *t* = 1.68).

Similarly, the volumetric assessment of the CC (Fig. 5*N*) at the level of the transplantation site, from bregma –1.5 to +1.5 mm, showed a volume decrease of the right injured CC (63.93 ± 2.21%, 2-way ANOVA, followed by Bonferroni’s test, *F*(2, 34) = 228.6) compared to the uninjured contralateral CC (normalized at 100, *p* < 0.0001, *t* = 24.18) or to the ipsilateral CC of the sham animals (104.08 ± 2.21% *p* < 0.0001, *t* = 27.40). After NPC transplantation in the CC, the volume of the right CC (103.15 ± 3.82%) was significantly improved compared to the vehicle group (63.93 ± 2.21%, *p* < 0.0001, *t* = 27.40) and became similar to control values (contralateral normalized at 100: *p* = 0.8822, *t* = 1.96; and ipsilateral sham: 104.80 ± 2.21%, *p* > 0.9999, *t* = 1.06). The corpus callosum is fully recovered after NPC transplantation.

#### HI injury trigger glial scar formation, and transplanted NPCs partially reduce gliosis in the CC

**Figure 6.**
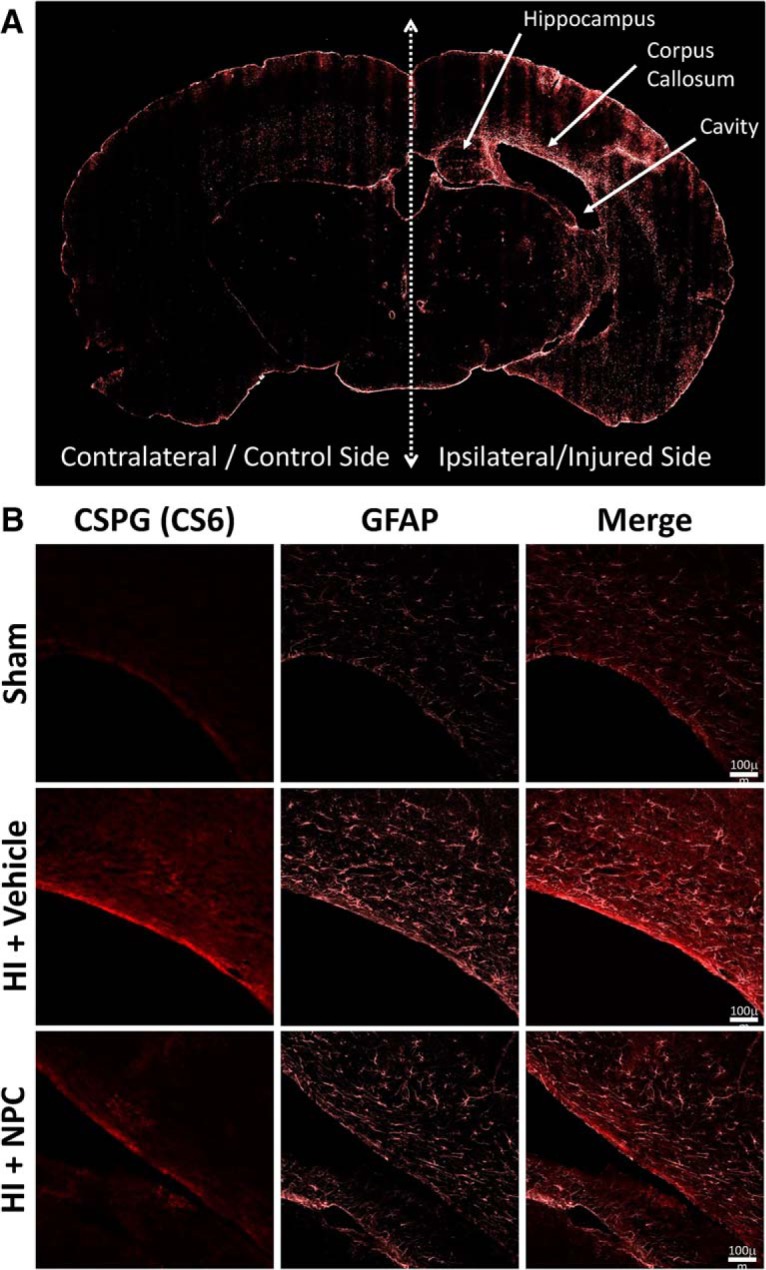
Transplanted NPCs do not significantly reduce glial scar formation. ***A***, Representative picture of a coronal brain section showing the left uninjured control side and the right HI injured side: GFAP staining is increased in several regions including the CC, the sensorimotor cortex, or the hippocampus. ***B***, Representative pictures of the CC at high magnification from control, HI + Vehicle and HI + NPC mice: the increased GFAP and CSPG staining levels show gliosis after HI injury, while a certain level of recovery is observed after NPC treatment.

The HI injury triggered gliosis in the injured hemisphere, which was observed mainly in the CC, the sensorimotor cortex, the hippocampus, and the internal capsule (Fig. 6*A*). Representative pictures at higher magnification show that the GFAP^+^ cell population, as well as the production of the glial scar component chondroitin sulfate proteoglycan (CSPG – CS6), were increased in the CC after HI. Importantly, following NPC treatment we observed a decrease in gliosis (Fig. 6*B*).

#### HI injury significantly affects the lateral ventricle, and transplanted NPCs lead to substantial recovery

**Figure 7.**
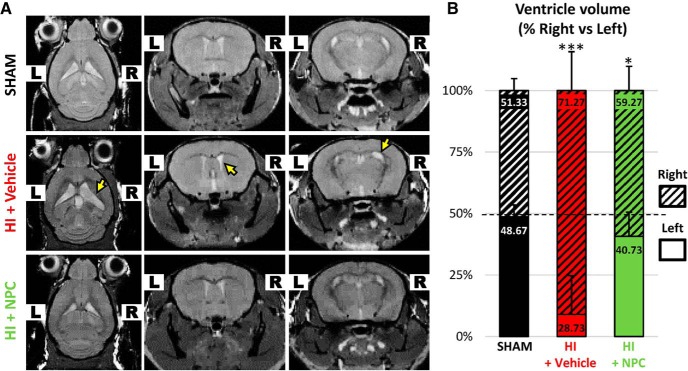
Transplanted NPCs help to recover the ventriculomegaly. ***A***, MRI shows right unilateral ventriculomegaly after HI injury (arrow) on axial sections (left column), coronal sections at the anterior brain level (middle column), and coronal sections at the posterior brain level (right column). ***B***, The percentage of right (dashed) versus left (plain) lateral ventricle showed a significant enlargement after HI (in red) compared to sham (in black). After NPC transplantation (in green) the brain structures, including the ventricle size, were partially restored (***A*** and ***B***). *p < 0.05, **p < 0.01, ***p < 0.001.

MRI analysis demonstrated clear unilateral damage to the right brain hemisphere following HI injury, with the right lateral ventricle (LV) being significantly enlarged compared to the control (Fig. 7*A*, arrows). Volumetric measurements (2-way ANOVA, followed by Bonferroni’s test, *F*(2, 36) = 15.55) showed that the right LV was dramatically enlarged (71.27 ± 12.34% of the total LV volume, i.e., right LV + left LV), compared to the contralateral LV (28.73 ± 12.34%, *p* < 0.0001, *t* = 8.36) and to the ipsilateral LV of sham animal (51.33 ± 4.83%, *p* = 0.058, *t* = 3.92). After NPC transplantation in the CC, the right ventricle size became smaller (59.27 ± 9.83%) than pretransplantation, although it remained larger than controls compared to the contralateral LV (40.73 ± 9.83%, *p* = 0.0127, *t* = 3.64) and to the ipsilateral LV of sham animal (51.33 ± 4.83, *p* > 0.9999, *t* = 1.56). The transplanted NPCs trigger a significant recovery of the ventricle volume (Fig. 7*B*).

### Transplanted NPCs lead to myelination

#### HI injury significantly decreased the OL population, whereas transplantation of exogenous NPCs increased the endogenous OL population, including mature OLs

**Figure 8.**
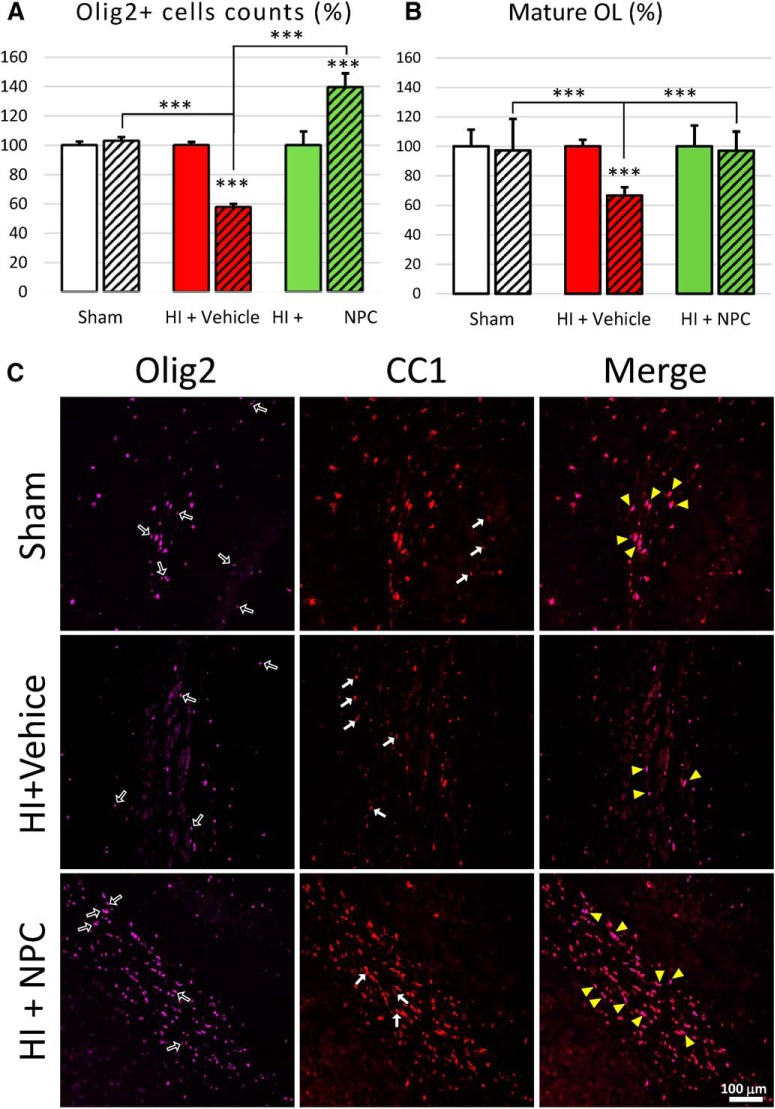
Transplanted NPCs have an impact on the endogenous oligodendrocyte population in the corpus callosum. Volumetric assessment of the oligodendrocyte population was performed around the sites of NPC transplantation (3-mm coronal virtual slice). ***A***, A significant decrease of Olig2^+^ cells was observed in the right CC after HI injury, while after transplantation, the Olig2^+^ cell population was dramatically increased. ***B***, The mature OL (Olig2^+^/CC1^+^) population was also impacted after HI injury and was restored after NPC treatment. ***C***, Representative pictures of immature (Olig2^+^/CC1^–^; black arrows) and mature (Olig2^+^/CC1^+^; yellow arrowheads) oligodendrocytes and astrocytes (Olig2^–^/CC1^+^, white arrows). The dashed versus plain bars represent the right versus left side, respectively. *p < 0.05, **p < 0.01, ***p < 0.001.

The endogenous OL (Olig2^+^) population (Fig. 8*A*) was significantly decreased after HI insult in the injured right CC (57.85 ± 2.19%, 2-way ANOVA, followed by Bonferroni’s test, *F*(2, 34) = 184.1) compared to the uninjured contralateral CC (normalized at 100, *p* < 0.0001, *t* = 14.37) or the ipsilateral CC of the sham animals (102.96 ± 2.54%, *p* < 0.0001, *t* = 15.38). After NPC transplantation, the Olig2^+^ cell population was markedly increased above the control level in the injured right CC (139.50 ± 9.43%, *p* < 0.0001, *t* = 12.47). Interestingly, the vast majority of those Olig2^+^ cells were also negative for the YFP-tag applied to NPCs.

The mature OL population (CC1^+^/Olig2^+^; Fig. 8*B*,*C*) was also affected by HI injury (2-way ANOVA, followed by Bonferroni’s test, *F*(2, 64) = 7.94). A decrease was observed (69.32 ± 6.74%) compared to the uninjured contralateral CC (normalized at 100, *p* = 0.0001, *t* = 4.88) and the ipsilateral CC of the control animals (97.20 ± 21.39%, *p* = 0004, *t* = 4.56). In HI + NPC animals, the mature OL population (97.05 ± 12.98%) was restored to uninjured levels and became similar to control values (contralateral normalized at 100: *p* > 0.9999, *t* = 0.63; and ipsilateral sham: 97.20 ± 21.391%, *p* > 0.9999, *t* = 0.03).

Fig. 8*C* shows representative images of Olig2^+^/CC1^–^ cells (immature OLs), Olig2^–^/CC1^+^ (astrocytes) and Olig2^+^/CC1^+^ (mature OLs). The transplanted NPCs lead to a major increase of the mature and immature OL population in the CC.

#### HI injury significantly affects the myelination of the CC, and NPCs lead to functional myelination

**Figure 9.**
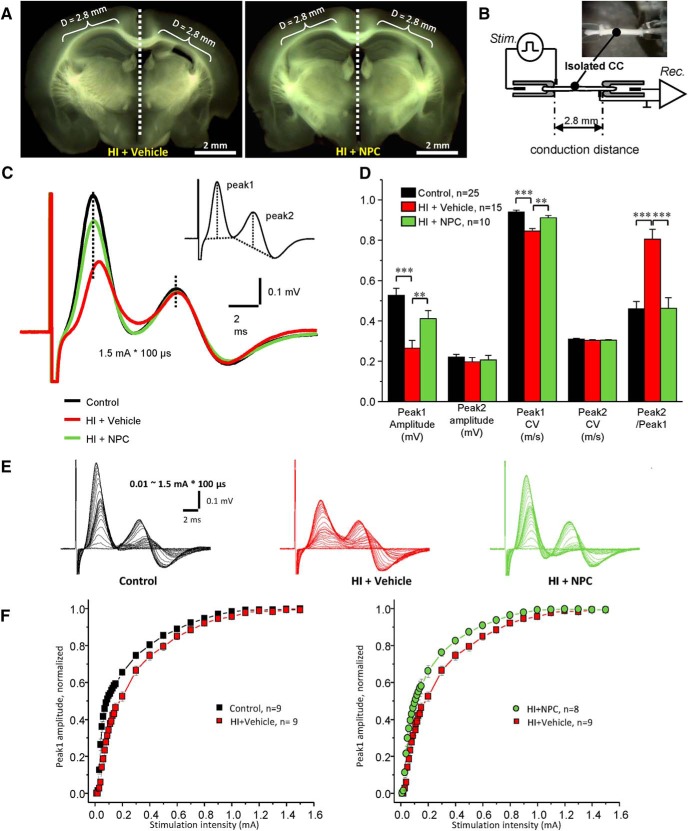
NPCs contribute to myelination after transplantation (electrophysiology). ***A***, Electrophysiological properties of the CC from HI + Vehicle (*n* = 15) and HI + NPCs (*n* = 10) mice: coronal slices (400 µm) of a HI (left side) and HI + NPCs (right side) from HI mice brains. ***B***, Diagram of the suction electrode recording method. The conduction distance of CC was 2.8 mm. ***C***, Averaged CAP traces recoded from left/control side of the CC (*n* = 15 + 10, black line), right/injured side of the CC (*n* = 15, red line) and right/injured side of the CC after NPC transplantation (*n* = 10, green line), respectively. Inset: the measurement of CAP amplitudes. ***D***, Detailed statistical comparison of CAP parameters from left/control CC (black bars), right/injured CC (red bars), and right/injured CC after transplantation (green bars). Conduction velocity (CV) calculated through the recording distance (2.8 mm) divided by the time between artifact and peak. ***E***, Relationship between stimulus intensities and amplitudes of CAPs: representative superimposed CAPs recorded at varying stimulus intensities (0.01∼1.5 mA) from left/control CC (left traces), right/injured CC (middle traces), and right/injured CC after NPC transplantation (right traces). ***F***, Comparison of the stimulus–response relationship of peak1 between left/control CC and right/injured CC (left plot, black and red, respectively); between right/injured CC with or without NPC treatment (right plot, red and green, respectively). All recordings were performed at room temperature. *p < 0.05, **p < 0.01, ***p < 0.001.

Functional myelination of the CC was assessed by electrophysiology. After unilateral HI injury, the amplitude of peak1 (myelinated axons) was significantly reduced in the right/injured side of the CC (0.27 ± 0.04 mV, *n* = 15) compared to the left/uninjured control side (0.53 ± 0.03 mV, *n* = 25, 1-way ANOVA, *p* < 0.0001, *t* = –5.14, *F* = 13.38), while the amplitude of peak2 (non-myelinated axons) was not significantly decreased in HI injured animals (Fig. 9*C*,*D*). Additionally, the CV of peak1 was dramatically attenuated in the right/injured side of the CC (0.84 ± 0.01 m/s, *n* = 15) compared to the left/control side of the CC (0.94 ± 0.01 m/s, *n* = 25, *p* < 0.0001, *t* = –6.2113, *F* = 19.37), but not in peak2 after HI (Fig. 9*C*,*D*). These results suggest that myelinated axons in the CC were dramatically degenerated and remained unmyelinated after unilateral HI injury, while nonmyelinated axons were not significantly affected. This data are supported by the significantly elevated peak2/peak1 ratio amplitude in the right/injured side of the CC (0.81 ± 0.05, *n* = 15) compared to left/control side of the CC (0.46 ± 0.04, *n* = 25, *p* < 0.0001, *t* = 5.79, *F* = 18.67; Fig. 9*D*). Fig. 9*E* shows superimposed CAPs recorded at varying stimulus intensities (0.01∼1.5 mA) from the left/control side, right/injured side of HI + vehicle mice and right/injured side of HI + NPC mice for the CC. Fig. 9*F* shows the relationship between stimulus intensity and CAP peak1 amplitude. Stronger stimulus intensities are required to generate CAPs in myelinated axons of the right/injured side of the CC compared to those of the left/control side of the CC (Fig. 9*F*, left plot). This suggests that larger myelinated axons are preferentially injured by HI.

After NPCs transplantation in the right/injured CC, the peak1 amplitude was significantly elevated (0.46 ± 0.04 mV) and was not significantly different anymore from the control (0.53 ± 0.03 mV, *p* = 0.8382, *t* = –1.09; Fig. 9*C*,*D*). The CV of peak1 CAPs also increased following NPC transplantation (0.91 ± 0.02 m/s) and became comparable to the control (0.84 ± 0.01 m/s, *p* = 0.3135, *t* = –1.65; Fig. 9*C*,*D*). The peak2/peak1 ratio amplitude was significantly reduced from 0.81 ± 0.05 (HI + vehicle) to 0.49 ± 0.04 (HI + NPC, *p* < 0.0001, *t* = –4.5933) and was not significantly different anymore from the control (0.46 ± 0.04, *n* = 25, *p* > 0.9999, t = 0.04; Fig. 9*D*). There were no significant effects of transplanted NPCs on peak2 of CAPs. The deficiency observed in HI animals with respect to stimulus intensity and peak1 amplitude was recovered after NPCs transplantation (Fig. 9*F*, right plot). CAP data suggest that after unilateral HI injury, NPCs significantly protect the myelinated axons and myelinate the unmyelinated axons, especially axons with a larger diameter.

### Transplanted NPCs lead to functional recovery

#### The cylinder test

**Figure 10.**
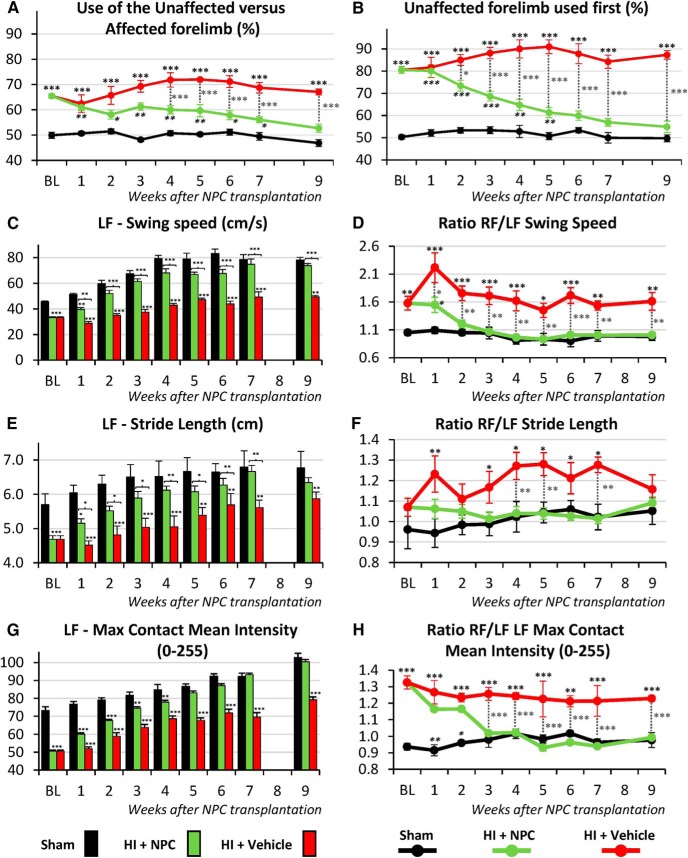
Transplanted NPCs improve functional recovery (cylinder test; gait analysis – CatWalkXT). ***A***, The HI mice used the unaffected right forelimb with a clear preference (cylinder test): the total use of the unaffected forelimb showed a clear unilateral impairment after HI injury. ***A***, The frequency at which the unaffected forelimb was used to start a rearing sequence also showed impairment. The NPC transplantation, as opposed to vehicle injection, led to functional recovery. ***C–H***, The HI mice showed significant impairment in several gait parameters such as swing speed (***C***, ***D***), stride length (***E***, ***F***), and paw intensity (***G***, ***H***) which were corrected after NPC transplantation. ***C***, ***E***, and ***G*** compare the left forelimb between groups, while ***D***, ***F***, and ***H*** compare the left versus right forelimb of each animals (ratio). BL = baseline. *p < 0.05, **p < 0.01, ***p < 0.001.

In our unilateral HI injury model only the ipsilateral/right brain hemisphere is injured, thus injured mice are anticipated to preferentially use the right unaffected forelimb (Fig. 10*A*,*B*). Before NPC transplantation at PND20, HI mice showed a clear preference to use the right forelimb for support during rearing exploration sequences (65.47 ± 0.77%, 2-way ANOVA, followed by Bonferroni’s test, *F*(2, 216) = 190.8) compared to control mice (49.92 ± 1.21%, *p* < 0.0001, *t* = 4.93). Following NPC transplantation, the preference for using the right forelimb gradually disappeared, and after 9 weeks, there was no forelimb use asymmetry in HI + NPC mice (52.69 ± 1.65%) compared to the control mice (46.83 ± 1.41%, *p* = 0.0551, *t* = 2.38; Fig. 10*A*). Conversely, the preferential use of the right forelimb persisted in the untreated, HI + vehicle mice (67.07 ± 1.19%) compared to the control mice (46.83 ± 1.41%, *p* < 0.0001, *t* = 6.42; Fig. 10*A*).

The choice of forelimb to start a rearing sequence also demonstrated functional asymmetry. Before NPC transplantation (PND20), the HI mice showed a clear preference for the right forelimb (80.51 ± 1.55%, 2-way ANOVA, followed by Bonferroni’s test, *F*(2, 216) = 336.1) compared to the control mice (50.32 ± 0.99%, *p* < 0.0001, *t* = 6.74). Following NPC transplantation, the right forelimb preference was reduced in HI + NPC mice, and after 9 weeks it was not significantly different compared to the control (54.91 ± 2.58% versus 49.75 ± 1.56%, *p* = 0.4257, *t* = 1.47; Fig. 10*B*). In contrast, HI + vehicle mice continued to use the right forelimb more frequently (87.31 ± 1.90%) than the control mice (87.31 ± 1.90% versus 49.75 ± 1.56%, *p* < 0.0001, *t* = 8.39; Fig. 10*B*). The cylinder test results following NPC transplantation demonstrate restored function in the affected forelimb.

#### The walking test

Brain subcortical and spinal cord networks mediate walking, and any alterations to these structures are known to impact walking performance. Several gait parameters were assessed to determine functional recovery following NPC transplantation. Here we present 1) swing speed (Fig. 10*C*,*D*), which is the speed of the paw while not in contact with the floor; 2) stride length (Fig. 10*E*,*F*), which is the distance between successive points of contact of the same paw; and 3) paw intensity (Fig. 10*G*,*H*), reflecting how the injury affects weight.

At baseline, the left/injured forelimb (LF) showed significant differences (2-way ANOVA, followed by Bonferroni’s test) in swing speed (33.19 ± 0.71 cm/s, *F*(2, 247) = 402.50), stride length (4.68 ± 0.11 cm, *F*(2, 247) = 73.50), and paw intensity (50.62 ± 0.59 intensity level 0–255, *F*(2, 247) = 581.30), compared to the left forelimb of the respective sham animals values (45.54 ± 0.84 cm/s, *p* < 0.0001, *t* = 4.43; 5.70 ± 0.32 cm, *p* = 0.0012, *t* = 3.60; and 73.28 ± 2.19, *p* < 0.0001, *t* = 13.43).

Following NPC transplantation, all 3 parameters showed recovery starting as early as 1 week after transplantation: swing speed (HI + NPC 39.53 ± 1.48 cm/s versus sham 51.27 ± 0.90 cm/s, *p* < 0.0027, *t* = 3.36), stride length (5.15 ± 0.13 cm versus 6.04 ± 0.23 cm, *p* < 0.0391, *t* = 2.50), and paw intensity (60.14 ± 0.70 versus 76.76 ± 1.52, *p* < 0.0001, *t* = 7.86).

After 9 weeks, there were no differences between HI + NPC and control groups in swing speed (73.76 ± 1.66 cm/s versus 78.06 ± 2.15 cm/s, *p* = 0.6604, *t* = 1.23), stride length (6.34 ± 0.14 cm versus 6.77 ± 0.48 cm, *p* = 0.6840, *t* = 1.21) and paw intensity (100.46 ± 1.29 vs 102.81 ± 2.39, *p* = 0.8029, *t* = 1.11; Fig. 10*C*,*E*,*G*). The lack of differences between the sham and HI + NPC groups at 9 weeks indicates a return of function to the left/injured forelimb of HI injured mice following transplantation of NPCs.

Similarly, the ratios of left/right forelimb values highlight the unilaterality of this functional impairment within each animal. Swing speed, stride length, and paw intensity ratios all showed significant impairment compared to sham animals, and these impairments were observed to disappear following NPC transplantation (Fig. 10*D*,*F*,*H*). Results from the gait analysis demonstrate that the transplanted NPCs contribute to functional recovery.

## Discussion

In this study, we first validated a hemiplegic neonatal hypoxia-ischemia model producing mild lesions, resulting in structural injury in various brain regions (e.g. cortex, hippocampus, CC) along with functional impairment. After NPC transplantation, partial structural recovery (of the cortex, lateral ventricle, and hippocampus) as well as full recovery (of the CC) were observed. We propose that the repair induced by exogenous NPCs is a nondirect effect involving the recruitment of endogenous oligodendrocytes to the lesioned site, leading to white matter repair.

### Our HI model mimics the clinical features of cerebral palsy

We generated a mild model of HI that displays pathobiological similarities to CP. One of the central features of CP pathology is WM injury, characterised here by the lesions observed in the CC. In addition, we observed in the ipsilateral/injured side a ventriculomegaly and an overall size reduction of the cortex, the hippocampus, and the brain hemisphere, which are consistent with past studies (Lubics et al., 2005; Lodygensky et al., 2010; Fatemi et al., 2011). Corroborating our data, clinical reports demonstrate that children with PVL exhibit an enlargement of the lateral ventricle (Davatzikos et al., 2003; Panigrahy et al., 2005), while periventricular regions, including WM, show structural and cellular abnormalities (Volpe, 2001, 2009b; Khwaja and Volpe, 2008).

The CC volume following HI decreased by an average of 36% and was in line with findings in previous neonatal animal studies (Sheldon et al., 1996; Skoff et al., 2001; Fatemi et al., 2011; Shrivastava et al., 2012). In many cases, the CC was found to be smaller in children with CP (Davatzikos et al., 2003; Panigrahy et al., 2005; Kulak et al., 2007; Ho et al., 2013; Andronikou et al., 2015).

The dysgenesis of the CC can be explained by the reduction of the OL population following injury and, specifically, by the significant decrease of mature OLs due to maturation arrest of the progenitors (Back et al., 2002; Segovia et al., 2008; Buser et al., 2010). The subsequent myelination impairment, due to reduced numbers of mature OLs, was confirmed by our histology (structural impairment, fewer mature OLs) and our electrophysiology data (functional impairment). Our findings showing reduced OLs and mature OLs are in line with clinical data, which confirm OL maturation disruption in children with CP (Buser et al., 2012; Back and Miller, 2014; Back, 2017). We demonstrated an association between CC impairment following HI and motor deficiencies. This is congruent with MRI studies that demonstrate a correlation between WM injury and severity of motor and cognitive impairment in children with CP (Panigrahy et al., 2005; Woodward et al., 2006; Skranes et al., 2007; Hollund et al., 2018). Furthermore, a negative relationship between the CC area and the Gross Motor Function Classification System has been observed (Kulak et al., 2007), confirming a link between the CC and motor function. Here, we demonstrated significant motor deficiencies using behavioral testing, which has previously been reported in similar animal models (van der Kooij et al., 2010; Braccioli et al., 2017). These impairments are in line with gait abnormalities observed in children with CP (Wren et al., 2005; Fonseca et al., 2018; Zhou et al., 2017; Davids et al., 2018; Mutoh et al., 2018). Taken together, these results demonstrate that our model reliably reproduces clinical features of CP.

### Transplanted NPCs survive, migrate, engraft, and morphologically differentiate in the corpus callosum

Following transplantation, we observed an NPC survival rate (∼4.5%) similar to those reported in a recent study from our lab (6.3%; Ruff et al., 2013b). Despite this low survival rate, we observed a significant effect on motor recovery. Interestingly, survival as low as 1% has been shown to be suitable for WM repair after spinal cord injury (Bottai et al., 2010).

The NPCs migrated and spread through the CC and reached the injured periventricular area. Interestingly, NPCs transplanted only in the left/uninjured CC migrated toward the right/injured CC and crossed the midline (data not shown). OLs/OL progenitors are known to migrate toward the site of brain injury (Gensert and Goldman, 1997; Nait-Oumesmar et al., 1999; Yandava et al., 1999; Skoff et al., 2001; Dizon et al., 2010; Obenaus et al., 2011).

NPCs have the potential to differentiate into OLs, neurons, and astrocytes *in vitro* (Karimi-Abdolrezaee et al., 2006; Eftekharpour et al., 2007). The differentiation profile observed here (OLs < 2%) was different than those reported in our previous studies: 97% of OLs in the uninjured *shiverer* mouse brain (Ruff et al., 2013b) and ∼80% in the spinal cord (Karimi-Abdolrezaee et al., 2010). Here we show that most of the transplanted NPCs remained positive for Nestin, a neural stem cell marker (Bernal and Arranz, 2018), suggesting that they have retained some NPC properties. We suggest that the specific *in vivo* heterogeneous microenvironment of the host tissue could explain this discrepancy.

However, the NPCs were anatomically well engrafted in the CC, where they differentiated morphologically, changing from a spherical to a fibroblast-like shape. While the transplanted NPCs did not notably colonize the neighboring structures, they grew numerous projections toward the cortex or the hippocampus. These morphologic changes suggest specific mechanisms of integration, with potential connections being made with the surrounding tissue via adhesion molecules, trophic factor release, or electrophysiological interactions (Nait-Oumesmar et al., 1999; Carmichael, 2003; Overman and Carmichael, 2014). Further, this engraftment is consistent with the “bio-bridge” concept, which relies on the establishment of a connecting framework by the transplanted stem cells between the lesioned site and the neurogenic subependymal zone (Sullivan et al., 2015; Lee et al., 2017; Liska et al., 2017). The endogenous cells involved in the repair process are recruited and guided by this bridge toward the injury site. Interestingly, once the bio-bridge is functional, the transplanted stem cells can disappear (Tajiri et al., 2013).

### Transplanted NPCs trigger cellular changes and result in structural recovery and functional improvement

Although the transplanted NPCs produced an effect on the ipsilateral/injured brain hemisphere and hippocampus, they failed to fully counteract the size decrease that arose after HI injury. Nevertheless, the ipsilateral ventriculomegaly was mostly restored, while the structural integrity of the CC was fully repaired. Likewise, after NPC transplantation in the CC, the OL population was restored and even increased above the normal level, as defined by the level of the contralateral side or the ipsilateral side of control mice, while the percentage of mature OLs was normalized. Furthermore, the lack of YFP staining of these cells confirms their endogenous origin.

In the dysmyelinated *shiverer* mouse, we have previously shown that NPCs differentiated to OLs at a rate of 97% and resulted in myelination (Ruff et al., 2013b). Here, by electrophysiology, we also demonstrated myelination of the unmyelinated axons of the injured CC. However, this appears to be performed by endogenous OLs attracted to the lesioned area by the Nestin-positive transplanted NPCs. The involvement of endogenous progenitors in myelination following a demyelinating injury is well established (Gensert and Goldman, 1997; Hawryluk et al., 2014; Buono et al., 2015), with precursors predominantly originating from subependymal zone cells, migrating toward the lesion site, and differentiating into OLs (Nait-Oumesmar et al., 1999; Etxeberria et al., 2010; van Tilborg et al., 2018). Alternatively, it has been proposed that endogenous OLs act locally, originating from the cortex or the striatum, with limited involvement from the subventricular zone (Dizon et al., 2010).

Our electrophysiology data confirmed that the CC myelination observed after NPC transplantation was functional. This corroborates the motor improvements posttransplantation observed in behavior testing, which showed a clear return of function for the impaired forelimb. This improvement following WM repair can be explained by the fact that the callosal motor fibers connecting the primary motor cortices of both hemispheres are central for bimanual coordination and learning of bimanual motor skill. Therefore, motor impairment following HI is likely due to a disconnection of the cortex from the midbrain, brainstem, and spinal cord following subcortical damage to descending fiber tracts and/or direct damage to the motor cortex (Clowry et al., 2014). For example, spastic CP is primarily a lesion of the corticospinal tract leading to secondary aberrant developments and the reorganization of movement and somatosensory cortical representations in the injured hemisphere (Jones and Adkins, 2015).

The white matter repair we have demonstrated here, as well as the functional myelination and the significant motor recovery, is most likely triggered by the increased endogenous OL population observed in the CC (+220% when comparing treated and untreated groups), resulting from a recruitment mediated/amplified by the transplanted exogenous NPCs. Interestingly, at the lesion site we have demonstrated glial scar formation, which is a dense structure consisting of hyperproliferative astrocytes that impede tissue regeneration (Khazaei et al., 2016; Ahuja et al., 2017). The reduction of the glial scar following NPC transplantation is likely to lower its inhibitive properties regarding regeneration and facilitate the migration of recruited endogenous cells for repair.

NPC survival rates as low as 1% have been previously shown to have an effect on WM repair (Bottai et al., 2010), supporting a nondirect effect mechanism involving a bio-bridge and/or trophic effect. Interestingly, NPCs are known to express molecules (e.g., platelet-derived growth factor-(PDGF)α, insulin-like growth factor (IGF)-1, brain-derived neurotrophic factor (BDNF), and erythropoietin (EPO) that are fundamental to oligodendrogenesis (Chicha et al., 2014; Buono et al., 2015; Yu et al., 2016) and could also improve brain repair via immunomodulation (Kokaia et al., 2012). Further, our lab has shown that *in vivo* transplantation of NPCs produces significantly higher levels of trophins such as IGF-1, nerve growth factor (NGF), leukemia inhibitory factor (LIF), ciliary neurotrophic factor (CNTF), epidermal growth factor (EGF), basic fibroblast growth factor (bFGF), and transforming growth factor (TGF)-β1, while lowering the expression of PDGF-α and vascular endothelial growth factor isoform (VEGF)-α (Hawryluk et al., 2012). These alterations demonstrate the environmental influence on the functioning of the NPC *in vivo*.

Taken together, the data presented here support the hypothesis that endogenous cells produce regenerative effects when triggered by exogenous NPCs (Dibajnia and Morshead, 2013; Dadwal et al., 2015; Yu et al., 2016; Ruddy and Morshead, 2018).

## Conclusion

Our model of mild, yet functionally significant, injury exhibits a pattern of lesions that is comparable with many aspects of clinical CP. We demonstrated that NPCs transplanted in the CC play a crucial role in recovery through the reduction of glial scarring and the attraction of endogenous oligodendrocytes, probably via a bio-bridge and/or trophic support. The WM repair observed here involves myelination. Future studies are needed to determine the origin of the recruited endogenous oligodendrocytes, as well as the potential trophic factors released by NPCs, and their cellular targets to further decipher the mechanisms underlying the recovery.

*Note Added in Proof:* The 7th author, Mohamad Khazaei, was incorrectly omitted from the list of authors in the Early Release version published October 22, 2018. The list of authors and author contributions has now been corrected in the copyedited version of the article.

## Synthesis

Reviewing Editor: Sunghee Cho, Weill Cornell Medical College at Burke Medical Research Insitute Decisions are customarily a result of the Reviewing Editor and the peer reviewers coming together and discussing their recommendations until a consensus is reached. When revisions are invited, a fact-based synthesis statement explaining their decision and outlining what is needed to prepare a revision will be listed below. The following reviewer(s) agreed to reveal their identity: NONE. Note: If this manuscript was transferred from JNeurosci and a decision was made to accept the manuscript without peer review, a brief statement to this effect will instead be what is listed below.

This manuscript from JN, which addresses the impact of NPC transplantation on structural and functional recovery in a hypoxic ischemic hemiplegic mouse model, has been evaluated by previous reviewers. Both reviewers concurred on the strengths of the study, considering the importance of the mouse model that has clinical features of cerebral palsy, technical soundness and rigor, and multidisciplinary outcome analyses including histological, anatomical and functional/behavioral assessments. The authors appropriately responded to major concerns in a cover letter and revised manuscript. For the lack of mechanism study, the authors provided white matter repair for a potential mechanism of NPCs' action. The authors also emphasized the inclusion of electrophysiological, immunohistochemical, and neurobehavior outcome assessments in this study in response to the issue on lacking ultrastructural and LM evidence of remyelination. In addition, there is an explanation for the intent of study, which was largely focused on motor functions and not in cognitive function assessment. The response was supplemented by a revised discussion on the bio-bridge concept and also included revised figures to demonstrate pathology.
